# Health Care Personnel’s Perspective on Potential Electronic Health Interventions to Prevent Hospitalizations for Older Persons Receiving Community Care: Qualitative Study

**DOI:** 10.2196/12797

**Published:** 2020-01-02

**Authors:** Martha Therese Gjestsen, Siri Wiig, Ingelin Testad

**Affiliations:** 1 Centre for Age-related Medicine Stavanger University Hospital Stavanger Norway; 2 Centre for Resilience in Healthcare Faculty of Health Sciences University of Stavanger Stavanger Norway; 3 Exeter University Medical School Exeter University Exeter United Kingdom

**Keywords:** health services research, community health services, hospitalization, health services for the aged, qualitative research, focus groups, eHealth, technology

## Abstract

**Background:**

The use of electronic health (eHealth) interventions is suggested to help monitor and treat degenerative and chronic diseases through the use of sensors, alarms, and reminders and can potentially prevent hospitalizations for home-dwelling older persons receiving community care. It is increasingly recognized that the health care personnel’s acceptance of a technological application remains a key challenge in adopting an intervention, thus interventions must be perceived to be useful and fit for purpose by the actual users.

**Objective:**

The aim of this study was to identify and explore the perspectives of managers and health care personnel in community care regarding the use of eHealth interventions in terms of prevention of hospitalizations for home-dwelling older persons receiving community care.

**Methods:**

A case study with a qualitative approach was carried out in community care in a Norwegian municipality, comprising individual interviews and focus group interviews. A total of 5 individual interviews and 2 focus group interviews (n=12) were undertaken to provide the health care personnel’s and managers’ perspective regarding the use of eHealth interventions, which could potentially prevent hospitalizations for home-dwelling older persons receiving community care. Data were analyzed by way of systematic text condensation, as described by Malterud.

**Results:**

The data analysis of focus group interviews and individual interviews resulted in 2 categories: potential technological applications and potential patient groups. Discussions in the focus groups generated several suggestions and wishes related to technical applications that they could make use of in their day-to-day practice. The health care personnel warranted tools and measures to enhance and document their clinical observations in contact with patients. They also identified patient groups, such as patients with chronic obstructive pulmonary disease or dehydration or urinary tract infections, for whom hospitalizations could potentially have been prevented.

**Conclusions:**

We have shown that the health care personnel in community care warrant various technological applications that have the potential to improve quality of care and resource utilization in the studied municipality. We have identified needs and important matters in practice, which are paramount for acceptance and adoption of an intervention in community care.

## Introduction

### Background

The global shift in demographics represents an epidemiological transition from a predominance of infectious diseases to noncommunicable diseases (ischemic heart disease, stroke, and chronic lung disease) [1]. The use of electronic health (eHealth) is suggested to help monitor and treat degenerative and chronic diseases through the use of sensors, alarms, and reminders [2-5].

The underlying assumption is that the use of digital technologies can potentially redesign care pathways in a way that will improve monitoring and treatment of degenerative and chronic diseases, encourage better self-management of health problems, and alert professional support if devices signal a problem [2,3,6-9], ultimately reducing the disruptive impact of acute unscheduled hospital admissions, for example, for older persons [10]. Previous research has identified that emergency hospital admissions often occur when an older person has reached a point of crisis because of a combination of circumstances, such as an exacerbation of a chronic condition, change in social setting, or a cascade of symptoms because of multimorbidity and frailty [11-17]. The use of eHealth could thus be applied as a tool to prevent a severe state of illness that requires hospitalization by discovering and addressing the patients’ symptoms at an early stage.

In addition, from a resource perspective, the prevention of hospitalizations for older persons has gained much attention in the last decade [14,15,18,19]. Persons aged older than 65 years are substantial consumers of hospital care; there is a peak in hospitalization rates for both men and women in the age group of 80 years and older in all European countries [20]. Increasing age is thus associated with an increasing demand for specialized health care [21-23], and this may threaten the sustainability of the health care systems, as a larger share of older persons in the population implies a dwindling proportion of the workforce, consequently likely to aggravate existing strains on formal health systems [24,25]. Norwegian policy documents emphasize that a major response to the resource challenge in health care is to enable and empower people to live in their own home for as long as possible, as well as provide timely treatment interventions at the proper level in the health care system. The government introduced the *Coordination Reform January 1, 2012*, which represents a transition in responsibility for providing health care services, where the municipalities are to play a much larger role in meeting the demand for services [26]. In the reform, the preventative perspective in health care is of great focus, where an important assumption is that there is a potential for preventing hospital admissions for the older persons receiving community care.

Despite the rhetoric associated with the benefits of adopting eHealth interventions in community care to prevent hospitalizations, the use of such technologies has not developed at the pace and scale anticipated [27]. The resource and safety challenges are appropriate and well-rehearsed incentives to adopt certain technology interventions, but it is increasingly recognized in the research literature that the health care personnel’s acceptance of the technological application itself remains a key challenge in adopting an intervention [28-30], underlining the vital importance that the involved stakeholders (eg, researchers, policy makers, health care personnel, patients, and carers) are able to judge the value of an eHealth intervention in its own right. Conversely, until we develop interventions that are considered to be useful and fit for purpose by the actual users, there will be reluctance regarding adoption of technologies in health care [30,31].

A review by Joseph et al [32] found that identifying issues and needs in practice were the main challenges related to the development and implementation of telehealth projects. This implies that identification of patients who might benefit from an intervention and a clearly defined role of a technological application (whether it is a new application, a new clinical tool, or a new system for delivering care remotely) are factors paramount for acceptance and adoption of an intervention [27,33]. These aspects are, however, not described in the body of research concerning the development of eHealth interventions in community care. Consequently, knowledge concerning the health care personnel and managers in this context of care is scarce.

On the basis of the notion that the managers and health care personnel in community care play a pivotal role in informing an eHealth intervention, it is of vital importance to explore their perspectives, thus gaining a better understanding of which technology-based interventions are deemed to be more appropriate and which patient groups an intervention could target. Ultimately, this knowledge can contribute to a more optimized intervention by increasing the probability for staff acceptance, as the intervention is developed on the basis of needs and suggestions defined by the managers and health care personnel in community care.

### Informing an Electronic Health Intervention in Community Care

EHealth interventions are suggested as a means to improve efficiency, quality, and safety of care [33,34]. According to previous research, the adoption and implementation of such interventions in a complex health care system is challenging. In complex systems, elements are interdependent and mutually reinforcing; they interact with other systems in unexpected ways, as they comprise several aspects, such as technologies, humans and its social environment, which can simultaneously be members of several interrelated systems [35,36]. This sociotechnical perspective recognizes that people, technologies, organizations, and process of care interact in complex ways [37-39]. The unique competence that the nurses are in possession of should be taken into account to optimize the uptake and use of an eHealth intervention. By nature, the intervention is intimately and reciprocally entwined with the professional skills and networks that support technology use and the development of community care services and with the local, national, and transnational policy on technological innovation and assisted living [37,40,41]. Thus, nurses who provide care using eHealth must be well-grounded in general nursing knowledge, theory, and practice competencies and should furthermore have clinical experience and capacity to possess attributes of intuition and creativity to enhance a holistic care [42].

On this backdrop, this paper focusses on building a rationale for adopting an eHealth intervention in community care by exploring the perspectives provided by the health care personnel and managers in community care. The UK Medical Research Council’s (MRC) framework for the development and evaluation of complex interventions [43,44] has guided the building of a rationale for adopting eHealth interventions in community care. The MRC’s framework is recommended for the development of interventions containing several interacting components. The study reported in this paper pertains to the first step in the framework, which is Development. It encompasses identifying a relevant existing evidence base, ideally by carrying out a systematic review [44]. However, components of an intervention can also be identified through focus group interviews with the patients or health care personnel [45].

### Aim and Research Questions

The aim of this study was to identify and explore the perspectives of the managers and health care personnel in community care about the use of eHealth interventions to prevent hospitalizations for home-dwelling older persons receiving community care.

There were 2 research questions that guided the study:

1. Which eHealth interventions do health care personnel identify as appropriate to apply to prevent avoidable hospitalizations of home-dwelling older persons receiving community care?
2. From the health care personnel’s perspective, for which patients could hospitalizations potentially be prevented?

## Methods

### Context

The study was carried out in an urban municipality in Western Norway. Community care in this municipality is organized into 4 geographically based units and comprises 1600 older persons. This study involved 2 of these units with 800 older persons receiving community care. The municipality was in the process of integrating eHealth solutions in community care during the next few years.

This study was undertaken as a work package (WP) in a larger project, *Development and Implementation of eHealth in Municipalities*. The WP reported in this paper aimed at (1) identifying relevant patient groups who could potentially take advantage of eHealth in community care, (2) identifying the health care personnel’s and managers’ perspective of and readiness to use eHealth in community care, and (3) based on findings in (1) and (2), suggesting an eHealth intervention for the case municipality.

### Design

The study design was a single embedded case study with a qualitative approach, comprising (1) individual interviews and (2) focus group interviews. A case study approach is particularly useful to employ when there is a need to obtain an in-depth appreciation of how eHealth could be used in community care to prevent hospitalizations for home-dwelling older persons, in its natural real-life context [46,47]. The case is defined as community care in a Norwegian municipality.

### Recruitment and Data Collection

A total of 5 individual interviews and 2 focus group interviews (n=12) were undertaken to provide the health care personnel’s and managers’ perspective regarding the use of eHealth interventions, which potentially could prevent hospitalizations for home-dwelling older persons receiving community care. We conducted 5 individual semistructured interviews with senior managers in the municipality, applying a semistructured interview guide that focussed on the potential use of eHealth in community care. Using purposeful sampling [48], we sought informants who were most able to inform us on the research question. Senior managers were selected because they held major roles in the municipality’s work with eHealth in community care and were in the best position to validate and provide relevant information for the study. Administrative personnel in the municipality who otherwise were not involved in this study recruited informants; they recommended potential informants who could best explicate the aspects of interest. MTG then asked potential informants face-to-face about participation and all accepted. There was no relationship between the informants and interviewer before study commencement. The interviews were conducted by the same person (MTG) for consistency and took place at the respective informants’ office, with only the informant and interviewer present. The interviews lasted approximately 60 min and were audiotaped and transcribed verbatim.

We used focus group interviews to explore the health care personnel’s perceptions of uptake and use of eHealth interventions that could potentially prevent hospitalizations. The focus group method is a useful data collection technique when the aim of the research is to explore attitudes, experiences, beliefs, and concerns [49]. In total, 2 focus group interviews (6+6 informants, n=12) were undertaken in 2014 by the author (MTG) as a moderator to ensure rich and relevant data [49]. A co-moderator made notes on observations and impressions during the interviews. Both interviews lasted approximately 90 min and were audiotaped and transcribed verbatim. A thematic interview guide was developed for the purpose of exploring aspects related to the uptake and use of eHealth interventions, including thoughts concerning which technological solutions they would have liked to have in their day-to-day practice and implementation and implications of eHealth in community care. To reduce the risk of any predetermined responses, participants did not see the interview guide before the interviews, thus also increasing the chance of open focus group discussions. To take advantage of homogeneity, shared experiences, and existing group dynamics, each focus group comprised health care personnel in direct patient care or nurse managers in community care. Administrative personnel in the municipality, who otherwise were not involved in this study, recruited informants. The studied municipality was in the process of integrating various technological applications in community care, consequently we were not in a position to seek participants who had operational experience of eHealth in their daily activities. To ensure appropriately experienced health care professionals working *on the ground*, we identified a maximum variation sample; 12 health care professionals were invited and all agreed. Of them, 11 women and 1 man in the age range between 30 and 55 years, who had worked in community care for more than 5 years, participated in the interviews.

### Data Analysis

Qualitative data were analyzed by way of systematic text condensation [50], as it is well-suited to analyze the multifaceted phenomena of eHealth. This approach involves the following steps in the analysis process: (1) establishing an overall impression of the data material and identifying preliminary themes, (2) identifying and sorting units of meaning into code groups, (3) condensing the contents of each of the code groups into subgroups, and (4) summarizing and recontextualizing the contents of each code group to generalize descriptions and concepts, in this case, related to the uptake and the use of eHealth in community care. Malterud argues that the data analysis will benefit from being conducted by more than one researcher [50], thus all authors read all interview transcripts to get an overall impression of the full data material, (step 1 of the systematic text condensation process). This step of the analysis requires the researcher to read, with an open mind from a bird's-eye perspective, all pages with transcripts and then ask which preliminary themes (usually 4-8 themes) can be identified in the material. We identified 4 preliminary themes: factors related to implementation, ethical aspects, training, and potential use.

This paper reports on findings related to the theme *potential use* (an analysis of contextual factors related to implementation has been published elsewhere [51]). The first author (MTG) undertook all the subsequent data analysis pertaining to the *potential use* theme (steps 2-4 of the systematic text condensation process) with input from the coauthors. The analytical process is demonstrated in Table 1.

**Table 1:** Analytical process.

Meaning units (selected)	Subgroups	Categories
Dehydrated; they are admitted for a short time, have some IV and then sent home.	Short stay; Simple intervention	Identification of potential patient groups
COPD patients are left to themselves when they are discharged, and then the anxiety comes...	Discharged without support	Identification of potential patient groups
A lot of UTIs...many men who are catheterized for 1,5-2 litres. If we had a bladder scanner, we could have solved it ourselves...instead of going to the A & E.	Potentially preventable hospitalization	Identification of potential patient groups
We don’t have a bladder scanner, consequently we have to catheterize more often to be on the safe side, but then there is an infection and another hospitalization because of the infection	Use of technology to potentially prevent hospitalization	Identification of potential technological tool
A swollen leg, or whatever...there is much that could have been done if you could provide a picture or a video.	Video or photo as a tool for providing info about clinical condition	Identification of potential technological tool
... then we postpone, and eventually they are in such a bad shape that we have to call A & E.	—^a^	Identification of potential technological tool

^a^Not applicable.

### Ethics

This project has been approved by the Norwegian Data Protection Official (approval ref# 21/2013). Informants have provided a written consent with information that they could redraw from the study at any point and without reason. Qualitative data from the interviews were transcribed verbatim and anonymized by exchanging informants’ names with a number. We recorded informants’ gender and years of work experience. All data were collected and stored in accordance with data protection regulations; stored electronically on computers, which were access-controlled via passwords. Hard copies of transcripts were securely stored in locked filing cabinets in offices that were accessible only to research staff. Data will be deleted at the end of the study.

## Results

The data analysis of focus group interviews and individual interviews resulted in 2 categories: potential technological applications and potential patient groups. These 2 categories answer the research questions which eHealth interventions that are considered as appropriate to prevent hospitalizations, and a health care personnel’s perspective on which patient groups hospitalizations potentially can be prevented. Content from step (4) in the analysis (recontextualization) is presented as analytical text with category heading, respectively, and assembled with quotes that are representative of the category.

### Potential Technological Applications

Discussions in the focus groups generated several suggestions and wishes related to technical applications they could make use of in their day-to-day practice. Findings from the individual interviews identified several technology applications that could be useful in community care, but 1 manager expressed an important aspect:

It is very important to differentiate between the various types of technological applications; what can be useful in the day-to-day practice, for both the patients and the health care personnel in community care, in order to prevent hospitalizations and out-patient visits.

Head of health and social welfare department

The findings pertaining to this category demonstrates that the health care personnel warranted tools and measures to enhance and document their clinical observations in contact with patients. By doing so, they saw the potential of saving a trip to the outpatient emergency clinic for the patient and they could provide better quality of the home care.

In Norway, general practitioners (GPs) and doctors at the outpatient emergency clinic are obliged by law [52] to offer home visits to patients who are not fit to meet for a consultation at the doctor’s clinic or when it is deemed necessary to provide sufficient treatment and care. The focus group participants described a practice where home visits were seldom undertaken, because either way the patient had to go to the clinic to take the necessary tests. If, however, home care personnel could have done the tests, they would have saved the patient for a potentially strenuous transportation to the doctor’s clinic and at the same time reported much more precise clinical data. They had several suggestions in this matter; the possibility of drawing blood for a C-reactive Protein test (CRP) was suggested on the grounds that this was the first thing the doctor asked for when they made contact for an assessment of a patient. As they did not have the equipment to do this procedure, the patients had to book an appointment either with their GP or at the outpatient emergency clinic. According to the informants’ experience, this often also involved the use of an ambulance for transportation. One situation they described was when they would contact the outpatient emergency clinic (in night-time and/or weekends) and they could only provide a diffuse description of the patient’s condition, as they did not have access to measures that could help them be more precise in the description:

I’m calling the outpatient emergency clinic and report a patient who’s had a general decline throughout the week, and the personnel there say that we have to take a blood sample (C-reactive Protein=CRP) and oxygen saturation... we can’t perform this and consequently they are picked up by an ambulance.....We should have had the possibility to do these measures...

Several nurses, focus group interview 2

They also discussed the possibility of applying a video link to a doctor. This application could support their observations as well as provide a possibility for the doctor to assess a patient’s condition without being face-to-face. They suggested using video link as a tool for the doctor to observe symptoms related to respiration and swollen legs/peripheral edema.

Furthermore, the participants in the focus group interviews wished to be equipped in a manner that made them more self-sufficient in providing high quality care and suggested, for example, the use of a bladder scanner as a tool, in relation to a problem with reoccurring urinary tract infections (UTIs):

If we had a bladder scanner, we could have solved it ourselves...instead of going to the outpatient emergency clinic.

Nurse, focus group interview 1

This was discussed in the context of patients who had been scanned and catheterized several times per day during their hospital stay (because of UTIs), whereas when they were discharged from the hospital to their home, the home care personnel had no tools to help them observe the phenomenon of residual urine. This is a crucial observation for the prevention of UTIs [53].

Informants in both the focus group interviews and individual interviews suggested the use of a tablet in the day-to-day practice in community care. A tablet installed with the quality/record system used in community care would enable the health care personnel to enhance and document their clinical observations in contact with patients. To date of the data collection, the personnel documented the clinical assessments on paper, which they would plot once they came to the home care base (office) where they had access to a computer and the patients’ record. The informants expressed a clear potential to work safer, in terms of clinical measures being transmitted directly in the patients’ record, as contrary to first record the measures on paper, bring it to the home care base, and then manually plot them in the record.

In the focus group interviews, the use of a tablet was also discussed as a means to be more prepared when there was an emergency callout. Emergency callouts were a daily activity, as most patients had a safety alarm that would alert the health care personnel in community care if they activated it (ie, pushed a pendant alarm). A typical situation would be if a patient had fallen, but it could also be that they were tired of waiting for their medication or wanted help to get to the toilet. However, the health care personnel would only receive an alarm signal, and the first step in the response was to receive a phone call from an emergency dispatcher who provided information about the patient’s name, address, and phone number. If the alarm concerned a patient who they were not familiar with, they had no possibility to check the patient’s record for relevant information:

One is always out driving, on the way from one patient to another, and then you have to stop the car, receive information about which patient – their name, address and phone number, by phone and write it down. It would be much easier to receive a text message with this information, and then log on to the patient’s record on a tablet. I would like our quality system to be an app installed on a tablet!

Nurse, focus group interview 2

Informants both in the individual interviews and in the focus group interviews discussed technological applications related to a safe home environment and the potential for the patients to increase the degree of self-management using automated devices (smart house technology), alarms, and reminders. More concretely, they suggested that the safety alarm could be a hub for various types of applications, such as reminders for when to take their medication and when it was time to eat and movement-based light sensors located near the floor. The latter was suggested as a means to prevent patients from falling when they had to go to the toilet during the night.

### Potential Patient Groups

Findings pertaining to this category represent a direct response to the question about patients for whom hospitalizations could be prevented. The findings stemming from the individual interviews bear a notion of managers being motivated by national policy regarding the resource utilization. The managers did not talk about specific patient groups but had a more general approach to preventing hospitalizations for home-dwelling older persons, which they described to be an appropriate task for the municipality/community care to undertake.

We have to look at possibilities for how to follow up on home-dwelling patients—they should not be admitted to hospital! We should be able to draw blood in their home and do measurements in their home...

Assistant director

The informants in the focus group interviews started off by discussing various clinical conditions, and patients that they viewed did not necessarily need the competence provided in specialized health care services that a hospital represents. If a patient was to be hospitalized because of dehydration, they considered the *treatment* or intervention initiated at the hospital to be rather short and simple, implying that this sort of intervention did not require specialized health care:

Dehydrated patients; they are admitted for a short time, have some intravenous fluid (IV) and then sent home.

Nurse, focus group interview 2

Another group of patients who they discussed about was those who have Chronic Obstructive Pulmonary Disease (COPD). In their experience, these patients had frequent readmissions to hospital, not necessarily because of the clinical condition itself, but because of the anxiety that often follows having respiratory problems:

COPD patients are left to themselves when they are discharged, and then the anxiety comes...the use of a telemonitoring device for promptly measures is neat.

Nurse, focus group interview 1

The informants agreed that a clinical condition described as potentially preventable was UTI. Especially, male patients were characterized as vulnerable in this context, as the personnel had to perform what they described as excessive catheterizations for men who had problems with residual urine in the bladder:

A lot of UTIs...many men who are catheterized for 1,5-2 liters. We have to catheterize more often to be on the safe side, but then there is an infection and then they are hospitalized again due to this...

Nurse, focus group interview 1

As demonstrated above, the informants in this study are quite clear about which technological applications they consider potentially useful in their practice. They also discussed the patient groups for whom hospitalizations potentially could be prevented. There was partly a connection between the suggested technological applications and the identified patient groups. The results are discussed against the relevant literature, providing suggestions for future intervention research.

## Discussion

From a health care personnel’s perspective, the main incentive to adopt eHealth in community care was the practical use in daily care. The various technological applications as well as different patient groups were identified, where the use of technological applications potentially could provide a more precise clinical assessment of home-dwelling older persons receiving care services.

Findings from this study revealed that the health care personnel in community care were vigilant in observing clinical decline but lacked tools to measure this decline. More specifically, they actually warranted the use of technological applications in their work, implying that they regard the use of eHealth as integral to their nursing practice in community care. This perspective is in contrast to what May et al found in their study from 2011 [2], where they identified problems in terms of health care professionals in community care to be indifferent and sometimes even hostile to the implementation of telecare systems. In addition, a more recent study by Greenhalgh et al [4] found that some clinicians would adopt readily to the use of video outpatient consultations, whereas others needed incentives and support.

However, May et al [2] also found that some health care professionals adopted the telecare service regardless, given that they perceived it as effective. On the basis of interviews with potential users of eHealth solutions (ie, health care personnel), our findings suggest that such applications have the potential to enable the nurses in community care to provide a more accurate description of the problem(s) when contacting a doctor. This implies that there is a potential to increase the quality of community care through the use of warranted technological applications. Moreover, the likelihood of successful adoption is increased as the interventions are considered to be useful and fit for purpose by the actual users [2,31,32]. Thus, the approach applied in our study provides great value in terms of developing appropriate interventions to prevent hospitalizations for home-dwelling older persons receiving community care, as it identifies issues and needs in practice [27,32,54].

The latter aspect is extremely important as it is increasingly recognized that the health care personnel’s acceptance of the technological application itself remains a key challenge in adopting an intervention [28-30]. Furthermore, the informants in our study suggested the use of a video link to facilitate remote consultations with a doctor to deal with some of the nonurgent inquiries and potentially reduce the use of specialized health care services. Greenhalgh et al [4] found that video outpatient consultations appeared safe, effective, and convenient to use when the clinicians judged the patients to be clinically appropriate, but such situations were merely a fraction of the overall clinic workload. Although the use of a video link is perhaps not efficient in terms of reducing the workload, the informants in our study expressed an interest in saving patients for a potentially strenuous transportation to the doctor’s clinic. This is an important care aspect, even though it cannot compromise the appropriate health care interventions to be undertaken. The finding must be seen in concordance with the previous aspect; they saw the potential of providing improved quality of care, both in terms of making precise clinical assessments and caring for a patient´s resources. This holistic care practice is an expression of nurses who are well-grounded in general nursing knowledge, theory, practice competencies, and clinical experience and furthermore possess attributes of intuition and creativity to enhance a holistic care by the use of eHealth [42,55].

Previous research has identified that emergency hospitalizations of older persons often occur when the patient has reached a point of crisis, such as an exacerbation of a chronic condition, change in social setting, or a cascade of symptoms because of multimorbidity and frailty [14-17]. The use of technological applications as suggested by the health care personnel in our study could potentially prevent a severe state of illness that requires hospitalization, by discovering and addressing the patients’ symptoms at an early stage. This is important with regard to both quality of care and resource utilization [13,19].

### Limitations

This case study does not formulate a solution for how an eHealth intervention should be developed, but the insights from the study could inform a future intervention in comparable settings. One premise in this paper is to acknowledge that people and technologies are linked in a dynamic health care system made up of multiple interacting stakeholders. We have not focused on the patients or other stakeholders (eg, technology suppliers) as intended users of a technological solution. This needs to be explored for building an even more solid rationale for applying a technological application in community care. An intervention should be informed by all stakeholders—individual users, service providers, and technology suppliers— to ensure a person-centered, holistic, and ethical approach. Such coproduction should be addressed in future research.

The findings from this case study pertain to a particular community care and context prevailing in the included Norwegian municipality. Other municipalities, countries, and settings may illustrate different opportunities and challenges, which should be explored. It could be argued that our sample of informants including 17 community care managers and health care personnel should have been larger. However, based on the study’s rather narrow aim and the use of theory to extend the sources of knowledge beyond the empirical interview data, the sample offered sufficient information power, as described by Malterud et al [56]. The sample of 12 health care personnel had daily patient contact and represented future users of eHealth solutions. Hence, their perspectives may be transferable to other similar contextual settings as described in this study.

### Conclusions

Through this study, we have generated empirical knowledge about which eHealth interventions could potentially prevent hospitalizations for home-dwelling older persons receiving community care. By identifying issues and needs in practice we have identified factors paramount for acceptance and adoption of an intervention [27,54]. We have shown that the health care personnel in community care warrant various technological applications that have the potential to improve quality of care and resource utilization in the studied municipality.

Previous research has pointed to a poorly founded rationale for the use of an eHealth intervention as a reason for slow and fragmented uptake and use of eHealth in community care [27,41]. The findings in this study can specifically inform future interventions aiming to prevent hospitalizations for home-dwelling older persons in community care, as the identified potential applications are considered useful and fit for purpose. Furthermore, by providing a description of the development phase of a future intervention as described in the MRC’s framework [44], it adds significantly to the general body of knowledge regarding developing eHealth interventions in community care.

## Abbreviations

COPD: chronic obstructive pulmonary disease
CRP: C-reactive protein
eHealth: electronic health
GP: general practitioner
MRC: Medical Research Council
UTI: urinary tract infection
WP: work package
